# Ganciclovir and Its Hemocompatible More Lipophilic Derivative Can Enhance the Apoptotic Effects of Methotrexate by Inhibiting Breast Cancer Resistance Protein (BCRP)

**DOI:** 10.3390/ijms22147727

**Published:** 2021-07-20

**Authors:** Magdalena Markowicz-Piasecka, Johanna Huttunen, Ahmed Montaser, Santosh Kumar Adla, Seppo Auriola, Marko Lehtonen, Kristiina M. Huttunen

**Affiliations:** 1 Laboratory of Bioanalysis, Department of Pharmaceutical Chemistry, Drug Analysis and Radiopharmacy, Medical University of Lodz, ul. Muszyńskiego 1, 90-151 Lodz, Poland; magdalena.markowicz@umed.lodz.pl; 2 School of Pharmacy, Faculty of Health Sciences, University of Eastern Finland, P.O. Box 1627, FI-70211 Kuopio, Finland; johanna.huttunen@uef.fi (J.H.); ahmed.montaser@uef.fi (A.M.); santosh.adla@uef.fi (S.K.A.); seppo.auriola@uef.fi (S.A.); marko.lehtonen@uef.fi (M.L.); 3 Institute of Organic Chemistry and Biochemistry (IOCB), Czech Academy of Sciences, Flemingovo Namesti 542/2, 160 00 Prague, Czech Republic

**Keywords:** breast cancer resistant protein (BCRP), ganciclovir (GCV), methotrexate (MTX), MCF-7/MDA-MB-231 human breast cancer cells, multidrug resistance (MDR)

## Abstract

Efflux transporters, namely ATP-binding cassette (ABC), are one of the primary reasons for cancer chemoresistance and the clinical failure of chemotherapy. Ganciclovir (GCV) is an antiviral agent used in herpes simplex virus thymidine kinase (HSV-TK) gene therapy. In this therapy, HSV-TK gene is delivered together with GCV into cancer cells to activate the phosphorylation process of GCV to active GCV-triphosphate, a DNA polymerase inhibitor. However, GCV interacts with efflux transporters that are responsible for the resistance of HSV-TK/GCV therapy. In the present study, it was explored whether GCV and its more lipophilic derivative (**1**) could inhibit effluxing of another chemotherapeutic, methotrexate (MTX), out of the human breast cancer cells. Firstly, it was found that the combination of GCV and MTX was more hemocompatible than the corresponding combination with compound **1**. Secondly, both GCV and compound **1** enhanced the cellular accumulation of MTX in MCF-7 cells, the MTX exposure being 13–21 times greater compared to the MTX uptake alone. Subsequently, this also reduced the number of viable cells (41–56%) and increased the number of late apoptotic cells (46–55%). Moreover, both GCV and compound **1** were found to interact with breast cancer resistant protein (BCRP) more effectively than multidrug-resistant proteins (MRPs) in these cells. Since the expression of BCRP was higher in MCF-7 cells than in MDA-MB-231 cells, and the cellular uptake of GCV and compound **1** was smaller but increased in the presence of BCRP-selective inhibitor (Fumitremorgin C) in MCF-7 cells, we concluded that the improved apoptotic effects of higher MTX exposure were raised mainly from the inhibition of BCRP-mediated efflux of MTX. However, the effects of GCV and its derivatives on MTX metabolism and the quantitative expression of MTX metabolizing enzymes in various cancer cells need to be studied more thoroughly in the future.

## 1. Introduction

Multidrug resistance (MDR) is a common feature of many cancer types and a major challenge in clinical cancer treatment with chemotherapeutics [1,2,3]. In MDR, cancer cells have an intrinsic or acquired resistance towards a wide variety of anticancer drugs that significantly reduces the effectiveness of chemotherapy and most often also causes cancer recurrence. MDR may emerge from several mechanisms depending on the structure of anticancer agents. One of the most studied mechanisms is the over-expression of ATP-binding cassette (ABC) superfamily, drug effluxing transporters functioning on the plasma membrane, exemplified with historically the most significant efflux transporter, P-glycoprotein (P-gp, *ABCB1*) [2,4]. Later on, other efflux transporters, such as multidrug resistance protein 1 (MRP1, *ABCC1*) and breast cancer resistant protein (BCRP, *ABCG2*), also known as mitoxantrone resistant protein (MXR), were also associated with clinical MDR. Despite the extensive research with efflux transporter inhibitors, their success in clinical use has been modest, mainly because of the challenge of targeting these inhibitors only into the cancer cells [2,3,4,5].

Ganciclovir (GCV), a synthetic analog of 2′-deoxyguanosine, is an antiviral drug discovered in the 1980s that has been used against cytomegalovirus (CMV) infections [6,7,8]. To be active, GCV needs to be phosphorylated firstly to GCV-monophosphate (GCV-MP) by a viral thymidine kinase followed by subsequent phosphorylation to GCV-diphosphate (GCV-DP) and -triphosphate (GCV-TP) by ubiquitous cellular kinases. GCV-TP then inhibits DNA polymerase and thereby prevents viral DNA replication. The apoptosis-resulting effect of GCV has also been utilized in glioma therapy by introducing the herpes simplex virus thymidine kinase (HSV-TK) gene in an adenovirus-vector to remaining dividing cells after surgical removal of a brain tumor [9,10]. However, only a modest increase in median survival has been achieved in the clinical trials, most likely because of the poor targeting and delivery of the vector as well as GCV into these dividing cancer cells [9,11]. To solve the latter, it has been recommended that a more lipophilic derivative of GCV would be helpful, as GCV itself is very hydrophilic and less well penetrated across the cell membranes [12]. Moreover, it has been reported that GCV, in its non-phosphorylated form, is an MRP4-substrate, and therefore cancer cells overexpressing MRP4 are highly resistant to HSV-TK/GCV therapy [13].

In the present study, it was evaluated whether GCV and its more lipophilic novel derivative could serve as an efflux transporter inhibitors that could improve the outcome of another chemotherapeutic agent either in estrogen receptor-positive MCF-7 breast cells or triple-negative MDA-MB-231 breast cells. Thus, in this study, the effects of GCV and its more lipophilic derivative on methotrexate (MTX) cellular accumulation and subsequent apoptosis were explored, since MTX suffers from MRP1, 2, 3, and 4-related chemoresistance [14,15] and since it has been previously reported that MCF-7 and MDA-MB-231 cells overexpress MRP1/4 proteins [16,17]. Furthermore, the biocompatibility and effects of the novel derivative and GCV, as well as their combinations with MTX were evaluated in human plasma, erythrocytes, and endothelial cells (HUVEC). 

## 2. Results

### 2.1. Human Breast Cancer Cells Express Several Efflux Transporters

**Figure 1:**
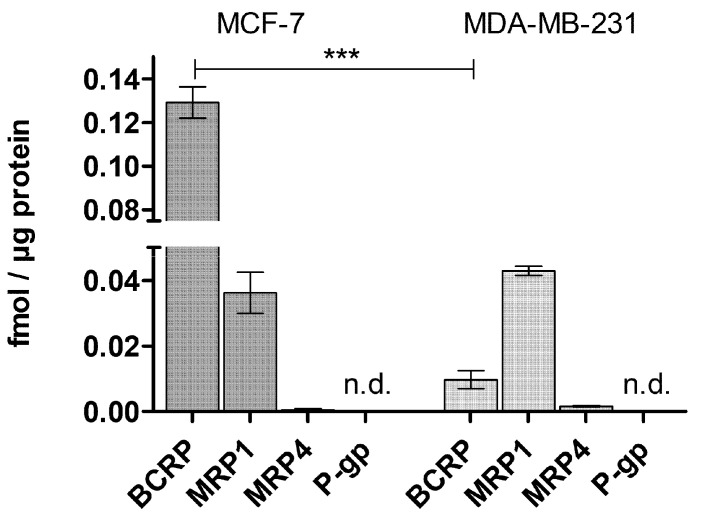
Quantitative protein levels of breast cancer resistant protein (BCRP), multidrug-resistant proteins 1 and 4 (MRP1 and MRP4), as well as P-glycoprotein (P-gp) analyzed from the plasma membranes of human breast cancer cells, MCF-7and MDA-MB-231 normalized to a housekeeping protein Na^+^/K^+^ATPase and the total amount of protein in the sample. The results are expressed as mean ± SD (*n* = 3) and an asterisk denotes a statistically significant difference, *** *p* < 0.001, one-way ANOVA, followed by Tukey’s test. The abbreviation n.d. denotes not detected (under the detection limit).

First, the protein expression of BCRP, MRP1, MRP4, and P-gp were quantified from the plasma membrane fractions of the selected human breast cancer cells, estrogen receptor-positive MCF-7 and triple-negative MDA-MB-231 (Figure 1). MCF-7 and MDA-MB-231 cell lines were chosen for this study based on the fact that they may have different expression profiles on efflux transporters, such as MRP 1 and 4, according to the previously reported Western blots [16,17]. In the present study, it was confirmed that in MCF-7 cells, BCRP expression was the highest (0.129 ± 0.007 fmol/µg protein), followed by MRP1 (0.036 ± 0.006 fmol/µg protein) and MRP4 (0.0005 ± 0.0001 fmol/µg protein). As a comparison, in MDA-MB-231 cells, the expression of MRP1 was highest (0.043 ± 0.001 fmol/µg protein), but curiously, at a similar range as in MCF-7 cells, followed by BCRP (0.010 ± 0.003 fmol/µg protein) and MRP4 (0.0015 ± 0.0002 fmol/µg protein). Notably, the quantifications of MRP4 were close to the lowest level of quantification (LLOQ), and therefore the numerical values of MRP4 need to be interpreted with caution. In addition, in both cell lines, the expression of P-gp was lower than the LLOQ, although it was detected in both cell types.

### 2.2. Synthesis of Lipophilic Derivative (1) of GCV 

**Scheme 1:**
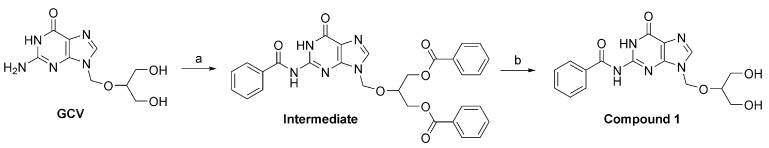
Synthetic route for the more lipophilic GCV-derivative **1**. Reagents and conditions: (**a**) Benzoyl chloride, pyridine, RT, overnight; (**b**) NaOMe, MeOH, RT, overnight, 47% (over 2 steps).

The more lipophilic derivative of GCV was prepared by coupling a benzoic acid to the free amino group of the guanine ring and hydroxyl groups of the alkyl side chain of the GCV to yield an intermediate that was subsequently treated with sodium methoxide to cleave the benzoyl groups selectively from the hydroxyl groups while leaving the amino-benzoyl group intact (Scheme 1). The formed compound **1** has also been previously described in the literature [18]. According to the calculated physicochemical properties, compound **1** is more lipophilic (log P raising from −2.54 to −0.31) while to topological polar surface area (tPSA) remained essentially the same (132.77 vs. 135.85) (Table 1).

### 2.3. GCV Can Effectively Accumulate into Human Breast Cancer Cells 

**Figure 2:**
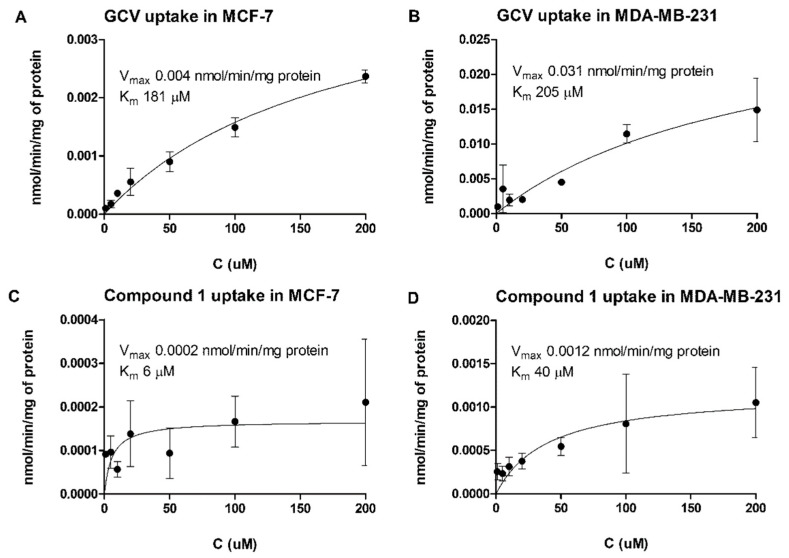
Cellular uptake of GCV into MCF-7 cells (**A**) and MDA-MB-231 cells (**B**) as well as compound **1** into MCF-7 cells (**C**) and MDA-MB-231 cells (**D**) over a concentration range of 1–200 µM (mean ± SD, *n* = 3–9).

Cellular uptake of GCV and its more lipophilic derivative (**1**) into MCF-7 and MDA-MB-231 cells were evaluated over a concentration range of 1–200 µM. The uptake of both compounds in both cell lines seemed to be concentration-dependent, implying that they were using transporters for their cellular accumulation (Figure 2). The maximum transport capacity (V_max_) of both compounds was significantly higher into MDA-MB-231 cells than MCF-7 cells (0.031 vs. 0.004 nmol/min/mg protein and 0.0002 vs. 0.0012 nmol/min/mg protein), pointing to an efficient efflux mechanism in MCF-7 cells. Furthermore, GCV uptake was more effective compared to the compound **1** (V_max_ of 0.0002 and 0.0012 nmol/min/mg protein in MCF-7 and MDA-MD-231, respectively). 

### 2.4. GCV-Derivative ***1*** Can Increase MTX Accumulation into MCF-7 Cells Most Likely via Interactions with BCRP

**Figure 3:**
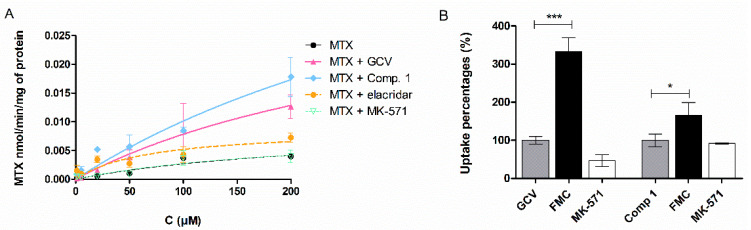
(**A**) Cellular uptake of MTX into the MCF-7cells over a concentration range of 1–200 µM (● filled black circles) and in the presence of 100 µM GCV (♦ filled blue diamonds) or compound **1** (▲ filled red triangles), 100 µM P-gp/BCRP inhibitor, elacridar (● filled orange circles with dotted line), and 100 µM multi-MRP-inhibitor, MK-571 (∇ unfilled green triangles with dotted line). (**B**) Cellular uptake of GCV and compound **1** (20 µM) into MCF-7 cells in the absence (gray bars) and presence of BCRP inhibitor, Fumitremorgin C (5 µM; black bars) of MRP-inhibitor MK-571 (100 µM; white bars). The results are expressed as mean ± SD (*n* = 3) and an asterisk denotes a statistically significant difference, * *p* < 0.05, *** *p* < 0.001, one-way ANOVA, followed by Tukey’s test.

Since MCF-7 displayed a greater efflux transporters’ expression profile than MDA-MB-231 cells, and GCV and compound **1** were most likely transported out of these cells due to their lower cellular uptake profile in MCF-7 cells, the cellular uptake of known efflux substrate, MTX (1–400 µM) was studied in the presence of GCV and compound **1** only with MCF-7 cells and compared to the situation without the compounds. As observed in Figure 3A, both compounds enhanced the cellular uptake of MTX; GCV by 21-times (V_max_ increased from 0.006 to 0.128 nmol/min/mg protein), and compound **1** by 13-times (V_max_ increased to 0.078 nmol/min/mg protein), while the positive controls P-gp/BCRP inhibitor, elacridar, and multi-MRP-inhibitor, MK-571, increased the uptake only moderately (V_max_ increased to 0.009 and 0.008 nmol/min/mg protein, respectively).

To find out whether GCV or its more lipophilic derivative **1** can have an affinity for the studied efflux transporters, their cellular accumulation (20 µM) into MCF-7 cells was studied in the absence and presence of BCRP-selective inhibitor Fumitremorgin C (FMC, 5 µM) and multi-MRP inhibitor MK-571 (100 µM). As shown in Figure 3B, the cellular accumulation of both compounds was significantly increased after pre-incubation of MCF-7 cells with FMC. Contrarily, no substantial effect was detected when the cells were pre-treated with MK-571. 

### 2.5. GCV and Its More Lipophilic Derivative ***1*** Can Improve Apoptotic Effects of MTX 

The effects of GCV and its more lipophilic compound (**1**) on the cell viability of MCF-7 cells were then determined by WST-1 assay. As the aim of the study was to evaluate if these compounds can potentiate the effects of MTX, the cell viability was determined first in the presence of the studied compounds alone. MTX (100 µM) inhibited the cell viability only by 29%, while GCV (50 and 100 µM) inhibited the cell growth by 5–11%, and compound **1** (50 and 100 µM) affected the viability by 8–18%. According to these values, it was selected that further apoptotic effects of these compounds were studied at 100 µM concentration and with 100 µM + 50 or 100 µM GCV or compound **1** combinations.

**Figure 4:**
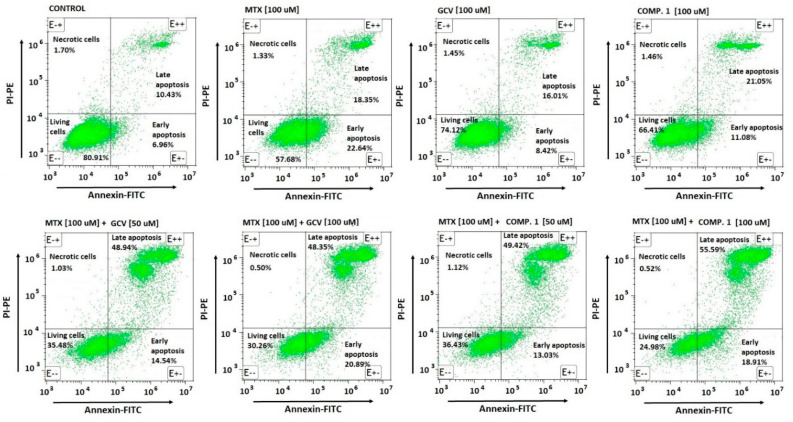
The effects of 100 µM MTX, GCV, or compound **1**, or their combinations (100 µM MTX+50 or 100 µM GCV or compound **1**) on the apoptosis of MCF-7 cells analyzed by Annexin V-FITC/PI double staining by flow cytometer.

The compounds and their mixtures with MTX were incubated with MCF-7 cells for 24 h and then stained with propidium iodide (PI) and Annexin V (AV) for flow cytometry analysis (Table 2, Figure 4). All the examined compounds were noticed to contribute to the significant decrease of the MCF-7 cell viability (10–19%) individually at 100 µM concentration, although to a similar but minor extent (*p* > 0.05, one-way Anova analysis). These effects were comparable to 10-times smaller concentration of a known anticancer agent, topoisomerase inhibitor etoposide (10 µM). The combination of GCV and MTX contributed to the greater decrease in the number of viable cells depending on the concentration of GCV (41% at 50 µM vs. 51% at 100 µM; Table 2). Importantly, these results were significantly lower than those obtained for MTX alone (19%). The combination of MTX and GCV resulted also in a significant increase in the percentage of early and late apoptotic cells (14–22% and 46–48%, respectively compared to 19% and 19% with MTX alone). Similar results were also obtained with MTX and compound **1** mixture (Table 2, Figure 4). The addition of compound **1** (50 and 100 µM) to MTX led to the significant and greater decrease in MCF-7 viability (46–56%) with a simultaneous higher increase in the percentage of early and late apoptosis (14–20% and 51–55%, respectively). Similarly to GCV, the effects of MTX with compound **1** were also concentration-dependent. 

### 2.6. Compound ***1*** and GCV Are Biocompatible in Human Plasma

**Figure 5:**
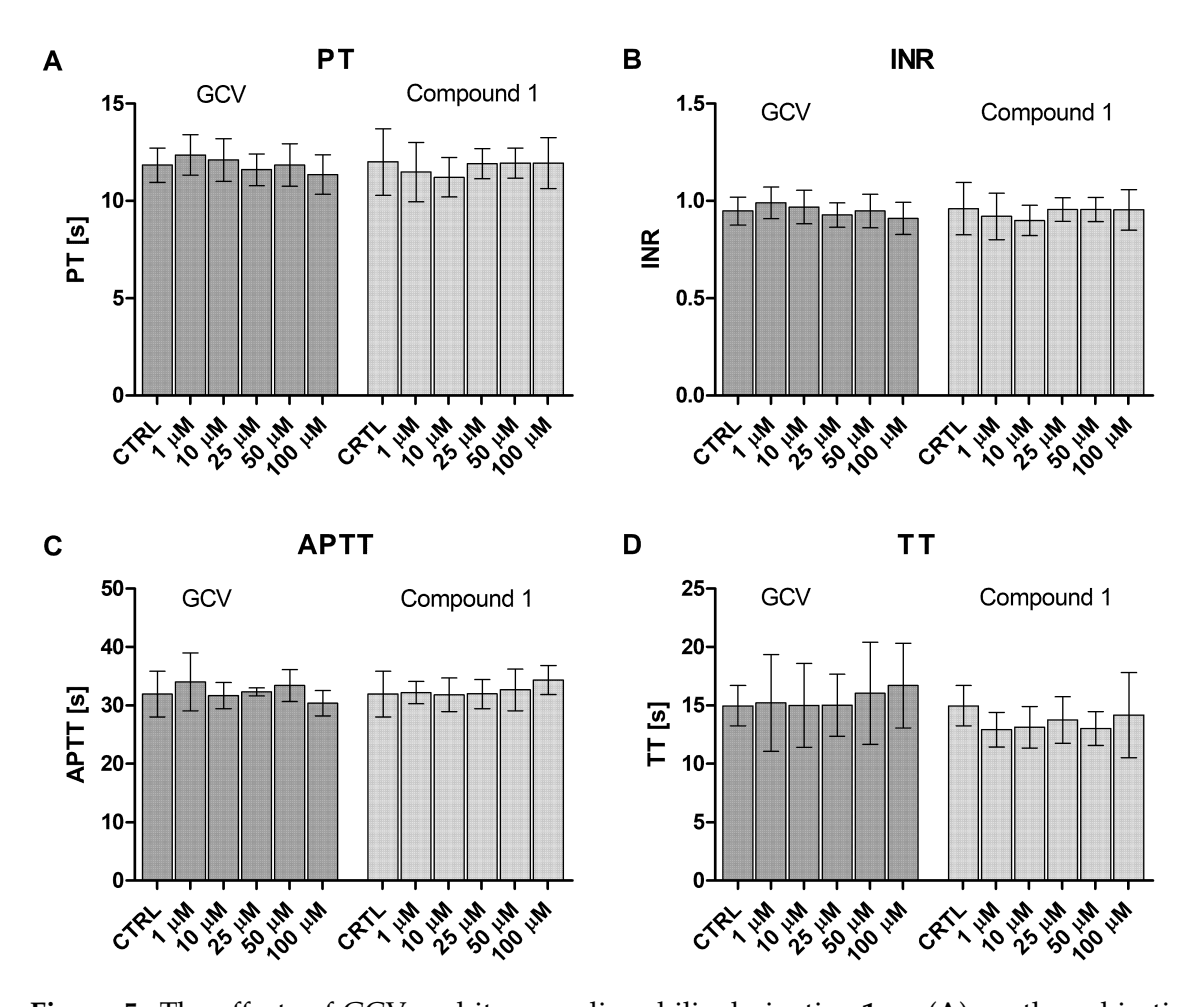
The effects of GCV and its more lipophilic derivative **1** on (**A**) prothrombin time (PT), (**B**) international normalized ratio (INR), (**C**) partially activated thromboplastin time (APTT), (**D**) and thrombin time (TT). The data are expressed as mean ± SD, *n* = 4–6.

In this study, the effects on the basic coagulation parameters of compound **1** were also screened to evaluate its biocompatibility and its potential to be used in humans. The results were compared to the ones of GCV. Neither GCV nor the lipophilic derivative **1** affected significantly any measured crucial plasma haemostasis parameters, including prothrombin time (PT), international normalized ratio (INR), partially activated thromboplastin time (APTT), and thrombin time (TT) over the studied concentration range (1–100 μM) (Figure 5) [19,20].

### 2.7. GCV and Compound ***1*** Do Not Affect HUVEC Viability

**Figure 6:**
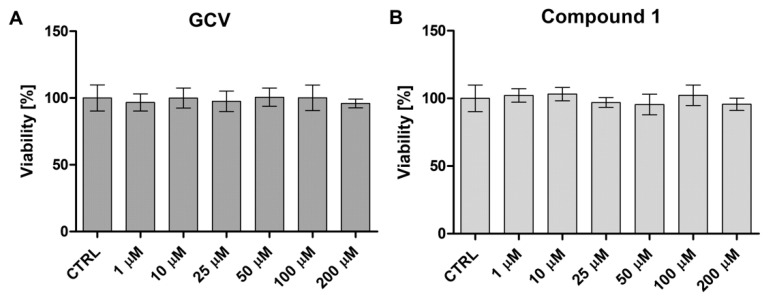
The effects of (**A**) GCV and (**B**) compound **1** on the viability of HUVEC. The results are presented as mean ± SD (*n* = 6–8).

The effects of GCV and its more lipophilic derivative **1** on the viability of endothelial cells (HUVEC) were also determined using WST-1 assay. Both compounds were found not to have any significant effects on the growth of HUVEC within the concentration range of 1–200 μM (Figure 6). 

**Figure 7:**
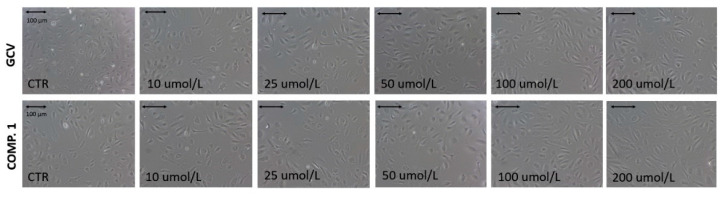
The effects of GCV and compound **1** on endothelial cell (HUVEC) viability at concentrations of 1–200 μM; cultures in medium alone were also used as controls. Representative phase-contrast cell images of control samples and cells treated with 10 and 200 μM of compounds are shown after 24 h of incubation (100-fold magnification). The scale 100 µm is indicated with black arrow.

Moreover, neither of the studied compounds contributed to the significant changes in HUVEC morphology at the concentration range 1–50 μM (Figure 7). In the case of 100 and 200 μM concentrations, slightly elongated cells could be observed with both compound treatments. In addition, HUVEC treated with 200 μM of GCV showed an increased number of small, bright dots in the field of view, which might suggest more cellular material after cell break down. However, such high concentrations are not clinically relevant, and therefore these compounds can be considered safe.

### 2.8. GCV and Compound ***1*** Can Affect Erythrocytes with High Concentrations

**Figure 8:**
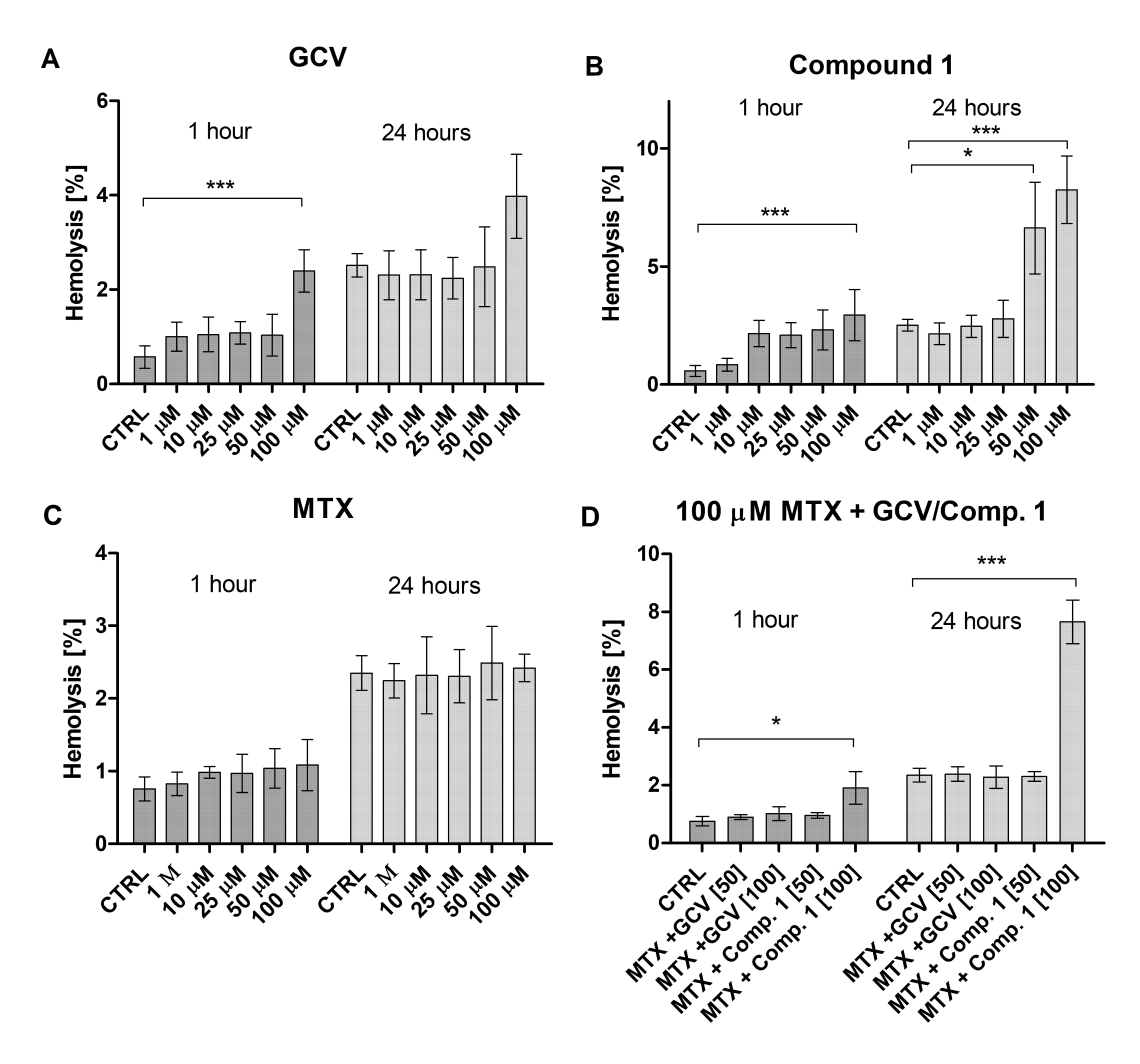
The effects of (**A**) GCV, (**B**) its more lipophilic derivative **1**, and (**C**) MTX (1–100 µM) as well as (**D**) combinations of MTX (100 µM) and GCV or compound **1** (50 and 100 µM) on the hemolysis rate after 1- and 24-h incubation. The results are expressed as mean ± SD (*n* = 4) and an asterisk denotes a statistically significant difference from the respective control * *p* < 0.05, *** *p* < 0.001, one-way ANOVA, followed by Tukey’s test.

The effects of GCV and compound **1** on the integrity of the erythrocyte membrane and subsequent hemolysis after 1- and 24-h incubation were also evaluated with a red blood cell (RBC) lysis assay. Curiously, 100 μM GCV was found to cause immediate interaction with RBCs manifested by a significant increase in the percentage of hemolyzed erythrocytes (2.40 ± 0.45% vs. control 0.57 ± 0.23% in Figure 8A). However, after 24 h of incubation of erythrocytes, the effect on RBC hemolysis diluted and the effect caused by GCV was not statistically significant compared to the control (3.98 ± 1.34% vs. 2.52 ± 0.25%). Similarly, GCV-derivative **1** contributed to the significant increase in hemolysis of erythrocytes. It appeared to exert a stronger effect on the disintegration of erythrocytes membrane once incubated for 24 h than GCV, since at 50 and 100 μM concentrations it induced hemolysis by 6.63 ± 2.54% and 8.25 ± 1.43% (*p* < 0.05) (Figure 8B).

Nevertheless, the effects of the most effective combinations with the chemotherapeutic agent of interest, MTX (in MCF-7 cells) were also evaluated in the erythrocyte model to assess their safety. MTX itself was found not to significantly affect the RBC membrane integrity over the studied concentration range after both 1- and 24-h incubation (0.83 ± 0.16–1.09 ± 0.35% vs. 0.76 ± 0.16% of control after 1 h incubation and 2.24 ± 0.24–2.49 ± 0.50% vs. 2.35 ± 0.24% of control after 24 h incubation) (Figure 8C). Importantly, MTX (100 μM) mixtures with GCV (50 or 100 μM) did not result in significant changes in the percentage of hemolysis (0.90 ± 0.08–1.02 ± 0.24% after 1 h incubation and 2.28 ± 1.03–2.39 ± 0.25% after 24 h incubation). Nevertheless, the combination of MTX (100 μM) with compound **1** (100 μM) contributed to the significant increase in the hemolyzed RBCs (7.65 ± 0.76% vs. 2.35 ± 0.24% for control, *p* < 0.001) (Figure 8D). However, when compared to compound **1** alone, the combination with MTX seemed to ameliorate these hemolytic effects, since with 50 µM compound **1** did not significantly differ from the control group (2.30 ± 0.17% vs. 2.35 ± 0.24%) unlike alone.

**Figure 9:**
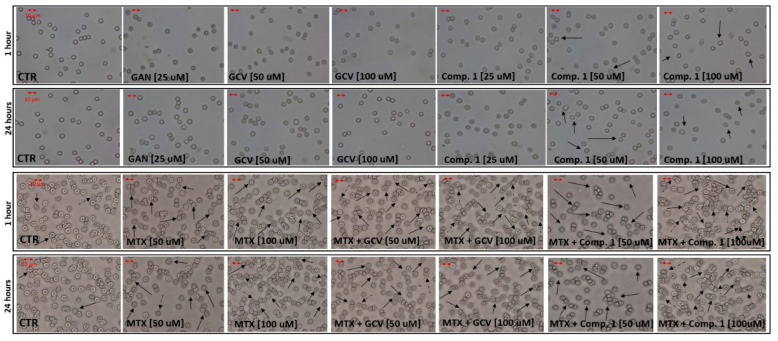
The effects of GCV and compound **1** (25–100 µM) as well as MTX (50 and 100 µM) and its combinations with GCV or compound **1** (50 or 100 µM) on erythrocyte morphology after 1 and 24 h incubation. Representative phase-contrast images are shown (magnification of 400 times) and the scale 10 µm is indicated with red color. CTR—control samples. Black arrows indicate echinocytes and black dashed arrows eryptotic erythrocytes.

The microscopic analysis of the erythrocytes showed that GCV did not influence the morphology of erythrocytes over the studied concentration range (Figure 9). Contrarily, compound **1** at 50 and 100 μM concentrations contributed to the formation of single echinocytes. Moreover, the analysis of erythrocyte morphology showed extensive echinocytosis in the samples treated with MTX (50 and 100 μM). Likewise, the studied combinations of MTX and GCV or compound **1** led to the formation of greater percentages of echinocytes and single eryptotic erythrocytes (Figure 9).

## 3. Discussion

In the present study, the quantitative proteomics revealed that there is no major difference in MRP1 and MRP4 expression levels among estrogen receptor-positive MCF-7 and triple-negative MDA-MB-231 cells (Figure 1), oppositely to the previous Western blot analyses [16,17]. However, a significant 13-fold difference was observed in BCRP expression between these cell lines, MCF-7 cells expressing BCRP to a greater extent than MDA-MB-231 cells. Previously, it has been reported that some sublines of MCF-7, such as MCF-7/AdrVp but not MCF-7 cells, express BCRP based on mRNA detection [21]. Moreover, it has been proposed that drug-sensitive MCF-7 and MDA-MB231 cells can be transfected with BCRP to resemble the phenotype of MCF-7/AdrVp. All in all, these quantitative proteomic results point out that it is highly important to recognize the expression profiles of efflux transporters in different cancer cell types that are responsible for the clinical MDR in order to find effective chemotherapy that may include a combination of specific efflux inhibitors. Furthermore, the expression of these transporters should be studied with highly sensitive methods, such as the quantitative proteomics method. However, it should be kept in mind that the expression of efflux transporters is inducible, and thus depends highly on the microenvironment of cancer cells.

It has been stated that the uptake of GCV into the rat glioma cells (BT4C) is not transporter-mediated, and because of the polar nature of GCV, the cellular (passive) permeation is poor [12]. Based on this ideology, we designed and synthesized more lipophilic derivative **1** of GCV (Scheme 1 and Table 1) and studied the cellular accumulation of GCV and the derivative **1** into MCF-7 and MDA-MB-231 cells. Curiously, the concentration-dependent uptake revealed that GCV was utilizing most likely some saturable transporter for its cellular accumulation. Furthermore, the maximum transport capacity (V_max_) of GCV into MDA-MB-231 cells was eight times greater than into MCF-7 cells (Figure 2A,B), indicating a possible efflux mechanism in MCF-7 cells. Similarly, derivative **1** showed a saturable transport mechanism, which was six times higher into MDA-MB-231 cells than MCF-7, although the cellular uptake in both breast cancer cells was much lower compared to GCV (Figure 2C,D). Indeed, it has been reported that GCV can use several distinct solute carriers (SLCs), including multidrug and toxin extrusion protein 1 (MATE1, *SLC47A1*) and 2-K (MATE2-K, *SLC47A2*), organic anion transporters 1 (OAT1, *SLC22A6*) and 2 (OAT2, *SLC22A7*), organic cation transporter 1 (OCT1, *SLC22A1*), as well as equilibrative nucleobase transporter 1 (EEG1, ENBT1, *SLC43A3*) [22,23,24,25]. Thus, based on the presented results, increasing the lipophilicity and size of a GCV-derivative decreased the transporter-mediated cellular uptake and is therefore not a feasible strategy to improve the delivery of GCV into the cancer cells. Instead, GCV derivatives should be designed based on the structure–activity relationships of the above mentioned transporters and the fact these transporters are overexpressed in target cancer cell types.

Both GCV as well as its more lipophilic derivative **1** were found to be biocompatible in human plasma and not causing any major effects on plasma haemostasis parameters (Figure 5). Moreover, neither of the compounds affected the viability of HUVEC (Figure 6 and Figure 7). However, both compounds were noticed to increases hemolysis at high concentrations (100 µM) and the hemolytic effects of derivative **1** were greater than those of GCV (Figure 8 and Figure 9). Therefore, the combination of MTX (100 µM) and compound **1** (100 µM) was also hemolytic, while GCV together with MTX can be considered a safer combination. GCV has been previously reported to be poorly tolerated in a combination of nucleoside reverse transcriptase inhibitor zidovudine in AIDS patients with cytomegalovirus infections. It has been suggested that MRP4 inhibition by GCV may have increased the cellular levels of zidovudine above cytotoxic levels. Therefore, a combination of GCV, a clinically approved drug, with any other compound can result in effective anticancer treatment, particularly where this primary chemotherapeutic suffers from efflux transporter-related chemoresistance. However, the levels of cytotoxicity in cancer cells vs. healthy cells need to be carefully studied to remain in a safe but effective therapeutic window.

Based on the above-mentioned cellular uptake results, it was explored if GCV or compound **1** can increase the cellular accumulation of the anticancer agent, MTX, since it has been reported that in addition to MRP1–4, MTX can interact also with BCRP [26,27]. In MCF-7, both compounds were able to improve the exposure of the cells to MTX, GCV increasing the MTX accumulation by 21-fold while compound **1** increased it by 13-fold (Figure 3A). It was also demonstrated in the present study that both GCV and its more lipophilic derivative **1** were interacting with BCRP, since a selective BCRP-selective inhibitor, FMC, was able to increase the amount of these compounds in the MCF-7 cells, when incubating FMC together with these compounds (Figure 3B). However, in this study, the conclusions were based only on the competitive cellular uptake studies, and thus more sophisticated methods, e.g., computational modeling, are needed to clarify the exact interactions between the compounds and BCRP [28,29,30]. Curiously, interactions of GCV and other anticancer nucleosides with BCRP have been demonstrated also in other studies [31,32], which supports our conclusions.

Moreover, it is highly important also to evaluate how the compounds affect the function and/or expression of BCRP in the future. Notably, the interactions of GCV or compound **1** with BCRP in the present study seemed to be stronger than with MRPs, since MK-571 (MRP inhibitor) did not increase their cellular uptake significantly. Nevertheless, the expression of MRP1 and 4 was lower than, e.g., the one of BCRP in MCF-7 cells, which may affect these conclusions. Therefore, more detailed affinity studies are needed to clarify this comparison between BCRP and MRPs in the future. It needs to be also remembered that we cannot exclude the possibility of GCV or compound **1** affecting MTX cellular accumulation by other means, such as increasing its metabolic stability, which also needs to be studied more thoroughly in the future. For example, MTX is polyglutamated by folylpolyglutamate synthase (FPGS), which increases the retention of MTX in the cells and thus MTX cytotoxicity [33,34]. On the other hand, γ-glutamyl hydrolase (γ-GH) removes these polyglutamates and thus predisposes MTX to efflux mechanisms. It is highly likely that the expression or function of these enzymes is affected by GCV and its derivatives.

The greater MTX exposure in the GCV or compound **1** combinations also resulted in lower cell viability in MCF-7 cells compared to the single treatments (MTX, GCV, or compound **1**). Accordingly, GCV as well as compound **1** contributed to the significantly lower number of living cells and a higher number of early and late apoptotic cells compared with the single treatment of MTX (Table 2, Figure 4). Curiously, there was no significant difference between the combination treatments, although GCV increased MTX exposure to a greater extent than compound **1**. Thus, considering the higher MTX accumulation and effects in MCF-7 cells after GCV or compound **1** co-treatments, which both had interactions with BCRP, it can be concluded that increased exposure of MTX is the most obvious explanation for the apoptotic effects seen with combination treatments. Moreover, the transport mechanisms of MTX (influx via reduced folate carrier 1 (RFC1) and efflux via several transporters, including MRPs and BCRP) have been stated to be the probable reasons for MTX resistance [34,35]. However, less focus has been paid to the metabolism of MTX. The cellular accumulation in this study was carried out with 30 min incubation, while in the apoptosis study, the cells were incubated for 24 h. Therefore, it is highly likely that in the MTX accumulation study, no metabolism was seen, while in the apoptosis study FPGS and γ-GH had enough time to affect MTX accumulation. Since the metabolism can differ significantly among different cancer cell types, these enzyme expressions and activities should be studied more thoroughly in the future.

## 4. Materials and Methods

### 4.1. Chemicals

All reagents and solvents used in these studies were commercial and high purity of analytical grade or ultra-gradient LC-MS-grade purchased from MilliporeSigma (St. Louis, MO, USA), J.T. Baker (Deventer, The Netherlands), Merck (Darmstadt, Germany), Riedel-de Haën (Seelze, Germany) or Thermo Fisher Scientific (Waltham, MA, USA), unless otherwise stated. Water was purified using a Milli-Q Gradient system (Millipore, Milford, MA, USA). The following reagents were used for basic coagulology screening, Bio-Ksel System APTTs reagent and calcium chloride (Bio-Ksel, Grudziądz, Poland), Bio-Ksel PT plus reagent (thromboplastin and solvent, Bio-Ksel, Grudziądz, Poland), and thrombin (3.0 UNIH/mL, Bio-Ksel, Grudziądz, Poland). Coefficients of variability were calculated using Bio-Ksel normal plasma (Bio-Ksel, Poland), and water for injection (Polpharma, Gdańsk, Poland). Triton X-100 used in the erythrotoxicity test was obtained from Polish Chemical Reagents (Gliwice, Poland).

### 4.2. Biological Material

The studies on the biological material were approved by the Bioethics Committee of the Medical University of Lodz (RNN/109/16/KE; 19 April 2016 and RNN/104/20/KE; 2 April 2020). RBCs for erythrotoxicity studies were separated from the plasma by centrifugation (3000× *g*, 10 min) at 20 °C and washed three times with 0.9% saline. Plasma was stored in small aliquots at −30 °C. Before the experiments, the material was thawed for 15 min at 37 °C.

### 4.3. Cell Cultures

MCF-7 human breast adenocarcinoma cells (HTB-22; RRID: CVCL_0031) was purchased from the American Type Culture Collection (ATCC, Manassas, VA, USA), MDA-MB-231 human breast adenocarcinoma cells (RRID: CVCL_0062) from Sigma-Aldrich (European Collection of Authenticated Cell Cultures (ECACC, Public Health England, Salisbury, UK, Cat. No 92020424), and human umbilical vein endothelial cells (HUVEC; RRID CVCL_2959) from Lonza (Basel, Switzerland, Cat. No. CC-2517). Both human breast cancer cell lines were cultured in standard conditions (37 °C, 5% CO_2_) using Dulbecco’s modified Eagle medium (DMEM, Gibco, Thermo Fisher Scientific, Waltham, MA, USA) supplemented with L-glutamine (2 mM, Gibco, Thermo Fisher Scientific, Waltham, MA, USA), heat-inactivated fetal bovine serum (10%, Gibco, Thermo Fisher Scientific, Waltham, MA, USA), penicillin (50 U/mL, Gibco, Thermo Fisher Scientific, Waltham, MA, USA), and streptomycin (50 μg/mL, Gibco, Thermo Fisher Scientific, Waltham, MA, USA). Once the cells reached 80% confluence in culture bottles (75 cm^2^), the cells were washed twice with DPBS solution (Gibco, Thermo Fisher Scientific, Waltham, MA, USA), and harvested using 5 mL of accutase (Sigma Aldrich, St. Louis, MO, USA). HUVEC were subcultured according to the manufacturers’ (Lonza, Basel, Switzerland) guidelines. Cell culturing ingredients for HUVEC included medium EGM-2 − medium + bullet kit (Lonza, Clonetics, Basel, Switzerland), accutase solution (Sigma Aldrich, St. Louis, MO, USA), and HEPES buffered saline solution (Lonza, Basel, Switzerland).

### 4.4. Protein Expression of Efflux Transporters in MCF-7 and MDA-MB-231 Cells

The absolute expression of the efflux transporters BCRP, MRP1, MRP4, and P-gp was quantified in the crude membrane fractions of MCF-7 and MDA-MB-231 cells by the LC-MS/MS method following a multiplexed multiple reaction monitoring (MRM) analysis mode according to the protocol described by Uchida et al. [36] with minor modifications. First, the crude membrane fractions were isolated from three distinct sets of cell culture plates using Membrane Protein Extraction Kit (BioVision Incorporated, Milpitas, CA, USA) according to the manufacturer’s instructions. The protein content for each fraction was measured by Bio-Rad Protein Assay, based on the Bradford dye-binding method (EnVision, Perkin Elmer, Inc., Waltham, MA, USA). A total amount of 50 µg protein from each fraction was solubilized/denatured in 7 M Guanidine hydrochloride, 0.5 M Tris-HCl and 10 mM EDTA-Na. The proteins were then reduced by dithiothreitol (1:50, *w*/*w*) and *S*-carboxymethylated by iodoacetamide (1:20, *w*/*w*) (Sigma-Aldrich, St. Louis, MO, USA). The alkylated proteins were precipitated by methanol/chloroform/water (4:1:3) and centrifuged at 18,000× *g* for 5 min at 4 °C. The pellet was resuspended in 6 M urea and mixed for 10 min at room temperature before the dilution with 0.1 M Tris-HCl (pH 8.5) to a final concentration of 1.2 M urea, and dissolved completely by intermittent sonication (Branson 3510, Danbury, CT, USA). The dissolved proteins were first digested with LysC (1/100, *w*/*w*) (MilliporeSigma, St. Louis, MO, USA) and 0.05% ProteaseMax (Promega Biotech AB, Nacka, Sweden) for 3 h at room temperature. Then, the samples were spiked with 10 µL (30 fmol) of the labeled peptides for absolute quantification (JPT Peptide Technologies GmbH, Berlin, Germany) (Table 1). The samples were incubated with (1/100, *w*/*w*) TPCK-Trypsin (Promega Biotech AB, Nacka, Sweden) for 18 h at 37 °C. The tryptic digestion was then quenched by adding 40 µL of 5% formic acid. The samples were then centrifuged at 18,000× *g* for 5 min at 4 °C, and the supernatants were transferred to vials for the analysis.

The digested peptides were analyzed using an ultra-performance liquid chromatography system coupled with a triple quadrupole mass spectrometer with a heated electrospray ionization source in the positive mode (UPLC 1290 and MSD 6495, Agilent Technologies, Santa Clara, CA, USA). A total amount of 20 µL of the digested peptides (10 µg) was separated using AdvanceBio Peptide Map 2.1 × 250 mm, 2.7 μm column (Agilent Technologies, Santa Clara, CA, USA) and LC eluents of 0.1% formic acid in water (A) and acetonitrile (B). The peptides were eluted following a constant flow rate of 0.3 mL/min and a gradient of 2–7% B for 2 min, followed by 7–30% B for 48 min, 30–45% B for 3 min, and 45–80% B for 2.5 min before re-equilibrating the column again for 4.5 min. The proteins were quantified based on the ratio between the light and heavy standard peptides, as described previously (Table 3) [37]. Data were acquired using Agilent MassHunter Workstation Acquisition (Agilent Technologies, Data Acquisition for Triple Quadrupole, version B.03.01) and processed by using Skyline software, MacCossLab, University of Washington, Seattle, WA, USA (version 20.1). The results were normalized to a housekeeping protein Na^+^/K^+^ATPase and expressed as fmol/µg of the total amount of protein in the samples.

### 4.5. General Synthetic Procedures

All reagents used in the synthesis of compound **1** were obtained from MilliporeSigma (St. Louis, MO, USA) or ThermoFisher Scientific (Waltham, MA, USA). Thin-layer chromatography with aluminum sheets coated with silica gel 60 F_245_ (0.24 mm) were used to monitor the reactions under UV and with a suitable coloring agent. Purifications were performed by flash chromatography on silica gel 60 (0.063–0.200 mm mesh). Nuclear magnetic resonance (NMR) spectra were recorded on a Bruker Avance 500 spectrometer (Bruker Biospin, Fällanden, Switzerland) operating at 500.13 MHz (^1^H) and 125.75 (^13^C), by using tetramethylsilane as an internal standard. Not all pH-dependent protons of the compounds were observed. ESI-MS spectra were recorded by an Agilent 1200 Infinity LC system coupled with an Agilent 6410 triple quadrupole mass spectrometer with an electrospray ionization source (Agilent Technologies, Palo Alto, CA, USA). Over 95% purity was confirmed by the UPLC system (Agilent Technologies 1290 Infinity II system; Agilent Technologies Inc., Wilmington, DE, USA), which comprised a high-speed pump, a multi-sampler, a multi-column thermostat (MCT), a diode array detector (DAD), and an Agilent ZORBAX Eclipse Plus C18 analytical column (2.1 × 50 mm, 1.8 µm) (Agilent Technologies Inc., Wilmington, DE, USA) eluting with water containing 0.1% formic acid (pH ca. 3.0) and acetonitrile containing 0.1% formic acid at the flow rate of 1.0 mL/min at room temperature.

### 4.6. N-(9-(((1,3-Dihydroxypropan-2-yl)oxy)methyl)-6-oxo-6,9-dihydro-1H-purin-2-yl)benzamide (1)

Ganciclovir (GCV; 0.10 g, 0.39 mmol) and benzoyl chloride (0.27 mL, 2.35 mmol) were stirred in anhydrous pyridine under Ar-atm at RT overnight. The solvent was removed and the residue was redissolved in CH_2_Cl_2_ (50 mL), washed with cold 3 M HCl (2 × 50 mL), saturated NaHCO_3_ (50 mL), and brine (50 mL), dried over Na_2_SO_4_, filtered, and evaporated to dryness under high vacuum. The intermediate was used for the next step without further purification.

The intermediate from the previous step was reacted with NaOMe (0.13 g, 2.34 mmol) in anhydrous MeOH under Ar-atm at RT overnight. The solvent was removed and the residue was redissolved in DCM and purified by a flash column chromatography eluting with MeOH:CH_2_Cl_2_ (0–20%) to yield an off-white solid, which was triturated with Et_2_O to give white solid 0.7 g (47% over 2 steps). ^1^H NMR (600 MHz, MeOD-d6) δ 8.23 (s, 1H, purine CH), 7.91–7.87 (m, 2H, benzene ring), 7.56–7.49 (m, 1H, benzene ring), 7.47–7.43 (m, 2H, benzene ring), 5.76 (s, 2H, NHC*H*_2_O), 4.08 (s, 2H, OH), 3.74 (tt, *J* = 6.3, 4.3 Hz, 1H, OC*H*(CH_2_OH)_2_), 3.51 (dd, *J* = 11.9, 4.2 Hz, 2H, OCH(C*H*_2_OH)_2_), 3.43 (dd, *J* = 11.9, 6.3 Hz, 2H, OCH(C*H*_2_OH)_2_). ^13^C NMR (126 MHz, DMSO-d6) δ 170.28 (amide CO), 163.57 (purine ring amide CO), 155.60 (N*C*(=)N), 149.52 (HN*C=*N), 139.67 (N=*C*N), 132.44 (benzene ring C), 132.28 (benzene ring C), 129.23 (benzene ring C), 128.48 (benzene ring C), 128.42 (benzene ring C), 128.24 (benzene ring C), 119.90 (C=*C*(N)CO), 80.40 (O-*C*H_2_-(CH_2_OH)_2_), 71.95 (N*C*H_2_O), 60.92 (2C, (O-CH_2_-(*C*H_2_OH)_2_)). MS (ESI^+^) for C_16_H_18_N_5_O_5_ (M+H)^+^: Calcd 360.35, Found 360.03. UPLC (254 nm) purity: 96.5%.

### 4.7. Cellular Uptake of GCV and Compound ***1***

For the cell uptake experiments, MCF-7 and MDA-MB-231 cells (passages 8–15) were seeded at the density of 1 × 10^5^ cells/well onto 24-well plates a day before the experiments. Cellular uptake of compounds was studied by incubating the cells at 37 °C for 30 min (uptake was linear with all compounds up to 30 min) with compounds at the concentration of 1–200 μM in pre-warmed HBSS buffer (250 μL). Subsequently, the cells were washed three times with ice-cold HBSS and lysed with 250 μL of NaOH (0.1 M) for 60 min. The lysates were diluted with acetonitrile (ACN) including the selected internal standard (labetalol) with a ratio of 1:3 and centrifuged at 10,000× *g* for 10 min. The samples were analyzed by liquid chromatography-tandem mass spectrometric (LC-MS/MS) methods with an Agilent 1200 Series Rapid Resolution LC System (Agilent Technologies, Palo Alto, CA, USA) together with an Agilent 6410 Triple Quadrupole Mass Spectrometer equipped with an electrospray ionization source by using a Zorbax Eclipse XDB-C18 column (50 mm × 4.6 mm, 1.8 μm, Agilent Technologies, Santa Clara, CA, USA).

The chromatographic separation of analytes was achieved with a gradient elution of water containing 0.1% (*v*/*v*) formic acid (A) and acetonitrile containing 0.1% (*v*/*v*) formic acid (B) at the flow rate of 0.50 mL/min, running with 15–40% B gradient for 0–0.5 min, 40–90% B gradient from 0.5 to 2 min, running isocratically 90% B from 2 to 5 min, B gradient return from 90% to 15% from 5 to 5.1 min, and finally the column was equilibrated with 15% B from 5.1 to 8 min. The column temperature was 40 °C and the injection volume was 5 μL. The following mass spectrometry conditions were used: electrospray ionization, positive ion mode; drying gas (nitrogen) temperature, 300 °C; drying gas flow rate, 6 L/min; nebulizer pressure, 25 psi; and capillary voltage, 4000 V. Analyte detection was performed using multiple reaction monitoring, the transitions being 256→152; 359.9→259 and 105; and 329→294 and 162 for GCV, compound **1**, and the internal standard (labetalol), respectively. Fragmentor voltages were 100 V for GCV and compound **1**, and 70 V for the internal standard. The collision energies were 10 V for GCV, 10 V, and 30V for compound **1**, and 10 V for the internal standard. Agilent MassHunter Workstation Acquisition software (Data Acquisition for Triple Quadrupole Mass Spectrometer, version B.03.01) was used for data acquisition, and Quantitative Analysis (B.04.00) software was used for the data processing and analysis. The lower limit of quantification (LLOQ) for the samples was 1.0 nM for compound **1** and 10 nM for GCV and the methods were linear selective, accurate (100 ± 5% of nominal concentration), and precise (RSD < 15%) over the range 1.0–2500 nM and 12.5–2500 nM, respectively. The concentrations of analytes in cell lysates were calculated from the standard curve that was prepared by spiking known amounts of compounds to ACN, including the selected internal standard and normalized with protein concentration. The protein concentrations on each plate were determined as a mean of three samples by Bio-Rad Protein Assay, based on the Bradford dye-binding method, using bovine serum albumin (BSA) as a standard protein and measuring the absorbance (595 nm) with a multiplate reader (EnVision, Perkin Elmer, Inc., Waltham, MA, USA).

### 4.8. Cellular Uptake of MTX

Cellular uptake of MTX was studied as described above for GCV and compound **1** at the concentration of 1–200 μM in MCF-7 cells (30 min incubation). The cell lysates were prepared accordingly and analyzed for MTX with LC-MS/MS (Agilent 1200 Series Rapid Resolution LC System together with an Agilent 6410 Triple Quadrupole Mass Spectrometer equipped with an electrospray ionization source) with a Zorbax Eclipse XDB-C18 column (50 mm × 4.6 mm, 1.8 μm, Agilent Technologies, Santa Clara, CA, USA) with a modified method that has been described previously [38]. The chromatographic separation of analytes was achieved with a gradient elution of water containing 0.1% (*v*/*v*) formic acid (A) and acetonitrile containing 0.1% (*v*/*v*) formic acid (B) at the flow rate of 0.30 mL/min, running with 5% B for 1 min, 5–90% B gradient from 1 to 5 min, 90–5% from 5 to 5.1 min, and finally the column was equilibrated from 5.1 to 8 min. The column temperature was 40 °C and the injection volume was 2 μL. The following mass spectrometry conditions were used: electrospray ionization, positive ion mode; drying gas (nitrogen) temperature, 300 °C; drying gas flow rate, 6 L/min; nebulizer pressure, 20 psi; and capillary voltage, 3000 V. Analyte detection was performed using multiple reaction monitoring, the transitions being 455 → 308 and 329 → 294, 162 for MTX and the internal standard (labetalol), respectively. Fragmentor voltages were 200 V for MTX and 70 V for the internal standard. The collision energies were 20 V and 10 V for MTX and the internal standard, respectively. Agilent MassHunter Workstation Acquisition software (Data Acquisition for Triple Quadrupole Mass Spectrometer, version B.03.01) was used for data acquisition, and Quantitative Analysis (B.04.00) software was used for the data processing and analysis. The lower limit of quantification (LLOQ) for the samples was 1.0 nM and the method was linear selective, accurate (100 ± 5% of nominal concentration), and precise (RSD < 15%) over the range 2.5–2500 nM. The concentrations of MTX in cell lysates were calculated from the standard curve that was prepared by spiking known amounts of compounds to ACN, including the selected internal standard and normalized with protein concentration. The protein concentrations on each plate were determined as a mean of three samples by Bio-Rad Protein Assay described above.

The cellular accumulation of MTX in the presence of GCV, compound **1**, P-gp/BCRP inhibitor, elacridar or multi-MRP-inhibitor MK-571 was carried out as described above. The cells were pre-incubated with 100 µM GCV, compound **1**, elacridar or MK-571 for 10 min and the incubation mixture was removed before adding the studied prodrug and LAT1 inhibitor on the cells. The uptake of 1–200 μM MTX (30 min) with 100 µM GCV, compound **1**, elacridar, or MK-571 was then carried out as the normal uptake. The concentrations of MTX were analyzed by the LC-MS/MS method described above and calculated from the spiked standard curve and normalized with the protein concentrations.

### 4.9. Accumulation of GCV/Compound ***1*** in the Presence of Efflux Inhibitors

The uptake of GCV and compound **1** in the presence of BCRP-selective inhibitor, 5 µM Fumitremorgin C (FMC), and multi-MRP-inhibitor, 100 µM MK-571, was studied as described above, by pre-incubating MCF-7 cells with FMC or MK-571 in HBSS buffer 10 min before adding the GCV or compound **1** solutions (20 µM) together with FMC or MK-571 on the cells and incubating them for 30 min. The amounts of GCV or its derivative **1** were analyzed by the LC-MS/MS method described above and calculated from the spiked standard curve and normalized with the protein concentrations.

### 4.10. Cell Viability

The viability of MCF-7 cells (cell passages 8–11) as well as HUVEC (passages 3–4) was determined in the presence of examined compounds (MTX, GCV and compound **1**) by using the WST-1 assay (Takara, Takara Bio Europe, Saint-Germain-en-Laye, France), which is based on the reaction of cleavage of tetrazolium salts by mitochondrial dehydrogenase in viable cells [39]. The cells were seeded at the density of 10,000 (MCF-7 and MDA-MB-231) or 7500 (HUVEC) per well on 96-well plates and maintained for 24 h to obtain 70% confluency, followed by treatment with the studied compounds: MTX (1–100 µM), GCV (1–100 µM) and compound **1** (1–100 µM) in MCF-7 and MDA-MB-231 cells, or GCV (0.1–200 μM) and compound **1** (0.1–200 μM) in HUVEC for 24 h (37 °C, 5% CO_2_). Control samples with pure medium and medium containing 10 µL of solvent (methanol+water) were performed to obtain 100% viability and the effects of solvent on cells, respectively. After 24-h incubation, the treating solutions were discarded, the cells were washed with culture medium (100 μL) and incubated with WST-1 reagent dissolved in the cell culture medium. The plates were incubated at 37 °C with 5% CO_2_ for between 1 (MCF-7 and MDA-MB-231) and 2 (HUVEC) hours and the absorbance was read at 450 nm using a microplate reader (iMARK, Bio-Rad, Bio-Rad Laboratories Inc., US). The results are expressed as a percentage of the control samples treated with pure medium, which constituted 100% viability. The effects of examined compounds on HUVEC were also evaluated by using an inverted microscope with phase-contrast (magnification 100×) (software OptaView 7, Opta-Tech, Warsaw, Poland).

### 4.11. Apoptosis Analysis

Apoptosis was examined using FITC Annexin V Apoptosis Detection Kit with propidium iodide (PI) (Biolegend, London, UK). MCF-7 cells were seeded at the density of 50,000 per well on 24-well plates, followed by 24-h incubation at standard conditions (37 °C, 5% CO_2_). Then, the medium was replaced by 250 μL of fresh medium (control wells) or medium with tested compounds or their combinations (100 µM MTX, GCV, compound **1** or 100 µM MTX+50 µM GCV or compound **1**, 100 µM MTX+ 100 µM GCV or compound **1**). Further, 10 µM etoposide was used as a positive control. The cells were incubated for an additional 24 h, followed by harvesting with accutase (Sigma Aldrich, St. Louis, MO, USA), collecting to Eppendorf tubes, and centrifuging (220× *g*, 5 min). The cell pellets were suspended in cold (4 ℃) cell staining buffer (BioLegend, London, UK) and washed once with this solution (220× *g*, 5 min). Then, the cells were resuspended in 100 μL of binding buffer (BioLegend, London, UK), and solutions of PI (10 μL) and FITC Annexin (5 μL) were added. The samples were vigorously vortexed and incubated for 20 min at room temperature in the dark. The analysis was conducted on the cytometer (CytoFlex, blue laser, 480 nm, Beckman Coulter, Brea, CA, US), and the results were analyzed using Kaluza 2.1 Beckman Coulter software. The analysis was performed according to the principle that annexin V (−) and PI (−) cells constitute living cells (E−−), annexin V (+) and PI (−) are early apoptotic cells (E−+), annexin V (+) and PI (+) are late apoptotic cells (E++), and annexin V (−) and PI (+) are necrotic cells (E−+). Moreover, 10,000 cells of each sample were analyzed, and the experiments were conducted in triplicate.

### 4.12. Basic Coagulation Tests (PT, INR, APTT, TT)

Coagulation parameters, including prothrombin time (PT), international normalized ratio (INR), partially activated thromboplastin time (APTT), and thrombin time (TT), were determined in the presence of the studied compounds (GCV and compound 1) according to the routine diagnostic procedure using a coagulometer (CoagChrom-3003 Bio-Ksel, Grudziądz, Poland) as described previously [40,41]. Control samples with distilled water and methanol mixture (1:1) were conducted in all tests. The methods were validated using normal plasma (Bio-Ksel, Grudziądz, Poland). Coefficients of variability for the tests were calculated; W_(PT)_ = 1.98%, W_(APTT)_ = 1.49%, W_(TT)_ = 1.15%. The reference values for each test equal PT: 9.7–14.6 s; APTT: 26.7–40.0 s; TT: 13.8–18.0 s for 3.0 UNIH/mL of thrombin.

### 4.13. Effects on Erythrocytes

The protocol for hemolysis assay has been previously described [38]. Briefly, red blood cell (RBC) suspension (2%) was prepared in 0.9% saline and incubated at 37 °C for one and 24 h with the tested compounds (GCV or compound **1**) at various concentrations ranging from 1 to 100 µM, or MTX (100 µM) or MTX + GCV or compound **1** (50 or 100 µM). The samples were then centrifuged (1000× *g*, 10 min), and the absorbance of the supernatant was measured at 550 nm (Cecil 2021, Cecil Instruments Ltd., Cambridge, UK). The degree of hemolysis was expressed as a percentage of released hemoglobin. Further, 2.0% *v/v* Triton X-100 was used as a positive control, which constituted 100% of hemolysis, whereas a sample of solvent (methanol + water) solution represented spontaneous hemolysis of RBCs (control). The coefficient of variability was counted: W_(1h)_ = 8.21%, *n* = 6; W_(24h)_ = 7.58%, *n* = 6.

For morphological evaluation, a 2% erythrocyte suspension was incubated with various concentrations of GCV or compound 1 for 1 and 24 h at 37 °C. Afterwards, the RBC morphology was evaluated using a phase-contrast Opta-Tech inverted microscope, at 400× magnification, equipped with software (OptaView 7, Opta-Tech, Poland) for image analysis.

### 4.14. Data Analysis

Physicochemical properties of the studied compounds were analyzed by ChemDraw Professional (PerkinElmer Informatics, Inc., Waltham, MA, USA, version 16.0.1.4 (77)) and MarvinSketch (ChemAxon, Ltd., Budapest, Hungary, Version 19.24.0) All statistical analyses, including the Michaelis–Menten kinetics of cellular uptake studies, proteomics, and IC_50_ values (the concentration of tested compound inhibiting cell growth by 50%) of the cell viabilities, and plasma biocompatibility (APTT, PT, TT) analyses were performed using GraphPad Prism v. 5.03 software (GraphPad Software, San Diego, CA, USA). Statistical differences between groups were tested using one-way ANOVA, followed by a Tukey’s multiple comparison test, and presented as mean ± SD, with significant difference denoted by * *p* < 0.05, *** *p* < 0.001. All the data are presented as mean ± SD (standard deviation) and the size of *n* varied among the study type (*n* = 4–9).

## 5. Conclusions

In conclusion, it was demonstrated in the present study that GCV and its more lipophilic derivative (**1**) can increase the cellular accumulation and apoptotic effects of MTX, most likely by inhibiting BCRP. These effects were seen in estrogen receptor-positive MCF-7 cells, due to the higher expression levels of BCRP, pointing to the importance of the quantitative determination of efflux transporter proteins on the plasma membrane of different cancer cell types. Although the cellular accumulation of GCV was higher into MCF-7 than that of more lipophilic derivative (**1**), their apoptotic effects were comparable. GCV and compound **1** were also uptaken more efficiently into triple-negative MDA-MB-231 breast cancer cells than MCF-7 cells, most likely due to the greater efflux interactions of compounds in MCF-7 cells. However, to improve the targeted delivery of GCV as an efflux inhibitor into the cancer cells, the utilization of cancer cell overexpressing GCV transporters, such as MATE1, MATE2-K, OAT1, OAT2, OCT1, and EEG1/ENBT1, is recommended to be explored. Moreover, the interaction with the metabolizing enzymes that are responsible for the effectiveness of chemotherapeutics are encouraged to be studied more thoroughly in the future. Lastly, this study points out that the usefulness and safety of new efflux inhibitors in combination with chemotherapeutics should be carefully studied in the early phase of the development of novel compounds.

## Figures and Tables

**Table 1:** Physicochemical Properties of GCV and Its More lipophilic Derivative **1**.

Property	GCV	Compound 1
Molecular weight (g/mol)	255.10	359.34
calculated log P	−2.54	−0.31
topological polar surface area (tPSA)	132.77	135.85
pK_a_	>14	>14

**Table 2:** Effects of 100 µM GCV and its derivative **1** alone and as combinations (50 or 100 µM) with 100 µM MTX on MCF-7 cells analyzed by Annexin V-FITC/PI double staining. 10 µM etoposide was used as a positive control. The results are presented as mean ± SD (*n* = 3).

Compound [μM]	Living Cells [E − −] [%]	Necrotic Cells [E − +] [%]	Early Apoptotic [E + −] [%]	Late Apoptotic [E + +] [%]
**Control**	79.6 ± 1.4	1.8 ± 0.2	8.0 ± 1.0	10.6 ± 0.3
**Etoposide** **[10 μM]**	65.9 ± 2.2 ***	1.8 ± 0.2 *	11.7 ± 1.5 **	20.5 ± 1.8 ***
**MTX** **[100 μM]**	60.5 ± 5.0 *	1.7 ± 0.3	18.6 ± 4.1 ^¥^	19.2 ± 2.3 *
**GCV** **[100 μM]**	69.9 ± 4.1 *	1.5 ± 0.3	10.6 ± 2.1	18.0 ± 1.9 *
**MTX + GCV** **[100 + 50 μM]**	38.7 ± 3.7 ** ^a^	1.1 ± 0.2 *	14.3 ± 0.2 *	45.9 ± 3.8** ^a^
**MTX + GCV** **[100 + 100 μM]**	29.4 ± 1.1 *** ^a^	0.5 ± 0.1 ** ^a^	22.3 ± 1.3 **	47.8 ± 0.8 *** ^a^
**Compound 1** **[100 μM]**	66.8 ± 0.8 **	1.5 ± 0.2	12.5 ± 1.2	19.3 ± 1.6 **
**MTX + Comp. 1** **[100 + 50 μM]**	34.1 ± 2.6 *** ^b^	1.4 ± 0.5	13.7 ± 1.4 *	50.8 ± 2.7 ** ^b^
**MTX + Comp. 1** **[100 + 100 μM]**	24.3 ± 2.0 *** ^b^	0.4 ± 0.1 ** ^b^	20.1 ± 2.1 **	55.2 ± 0.4 *** ^b^

MCF-7 cells were divided depending on staining with Annexin V and PI; (E − −) living cells; (E − +) necrotic cells; (E + −) early apoptotic cells; (E + +) late apoptotic cells. The values marked with asterisks represent statistically significant difference respective to the control (* *p* < 0.05; ** *p* < 0.01; *** *p* < 0.001). The values marked with (a) denote statistically significant (*p* < 0.01) difference between MTX (100 µM) respective to MTX and GCV combinations. The values marked with (b) denote statistically significant (*p* < 0.01) difference between MTX (100 µM) respective to MTX and compound **1** combinations. ¥—*p* = 0.064.

**Table 3:** SRM/MRM transitions for absolute quantitative proteomics.

Protein	Gene	Peptide	Type	Retention Time	Precursor Ion	Product Ions		
					Q1	Q3-1	Q3-2	Q3-3
Na^+^K^+^ATPase	*ATP1A3*	AAVPDAVGK	St	10.8	414.23	685.39	586.32	489.27
		AAVPDAVGK *	SIS	10.8	418.24	693.40	594.33	497.28
BCRP	*ABCG2*	SSLLDVLAAR	St	41.8	522.80	757.45	644.37	529.34
		SSLLDVLAAR *	SIS	41.8	527.81	767.46	654.38	539.35
MRP1	*ABCC1*	TPSGNLVNR	St	9.8	479.26	759.41	672.38	388.23
		TPSGNLVNR *	SIS	9.8	484.26	769.42	682.39	398.24
MRP4	*ABCC4*	APVLFFDR	St	37	482.77	796.44	697.37	584.28
		APVLFFDR *	SIS	37	487.77	806.44	707.38	594.29
P-gp	*ABCB1*	NTTGALTTR	St	28.8	467.75	561.33	490.29	377.21
		NTTGALTTR *	SIS	28.8	472.75	571.34	500.30	387.22

R * = Arg U-13C6; U-15N4, K * = Lys U-13C6; U-15N2, St: Standard Peptide, SIS: Stable Isotope Standard Peptide.

## Data Availability

Data sharing is not applicable to this article.
